# Supplementary figures and images for: Mitigating polyethylene-mediated periprosthetic tissue inflammation through MEDSAH-grafting
Source: PLoS One. 2024 Jun 6;19(6):e0301618. doi: 10.1371/journal.pone.0301618 (PMC11156361; doi:10.1371/journal.pone.0301618)

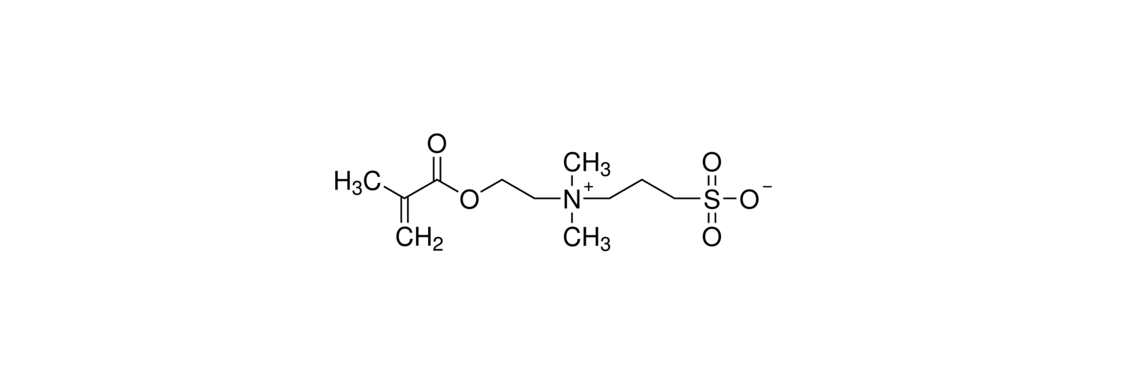

Supplement: S1 Fig — (TIF) [file pone.0301618.s001.tif]

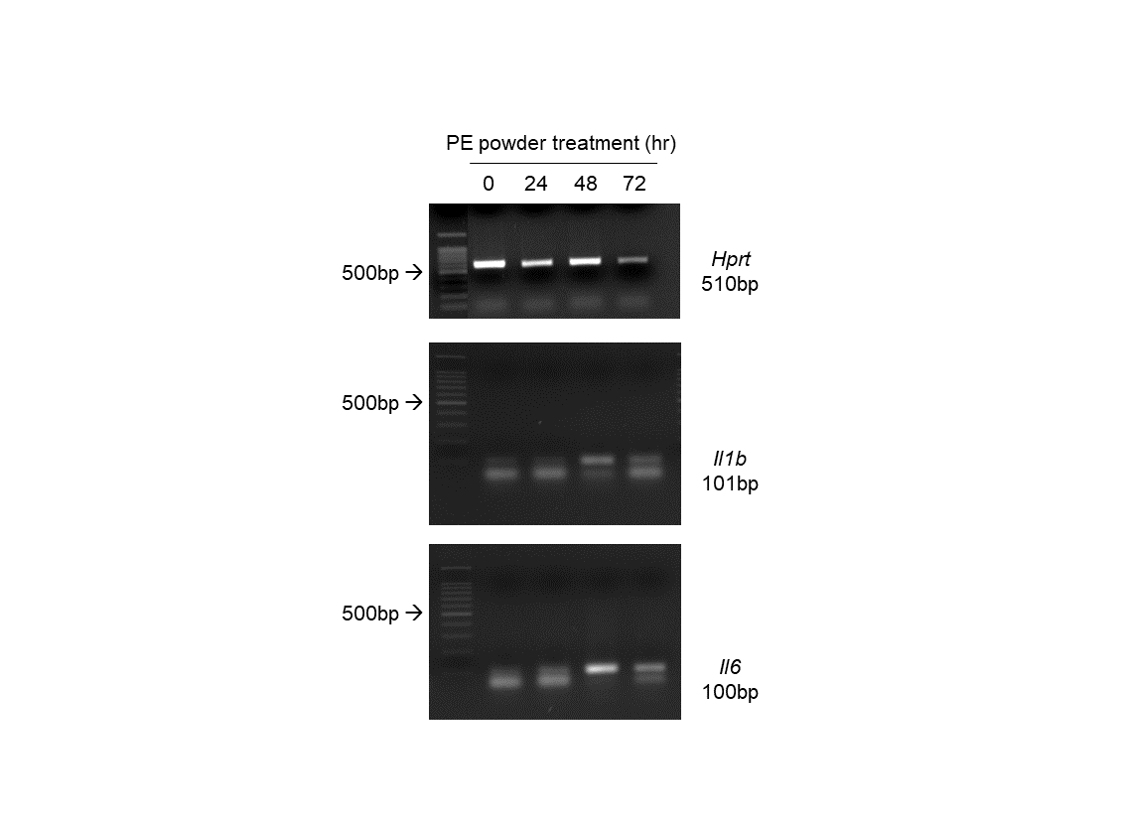

Supplement: S2 Fig — (TIF) [file pone.0301618.s002.tif]

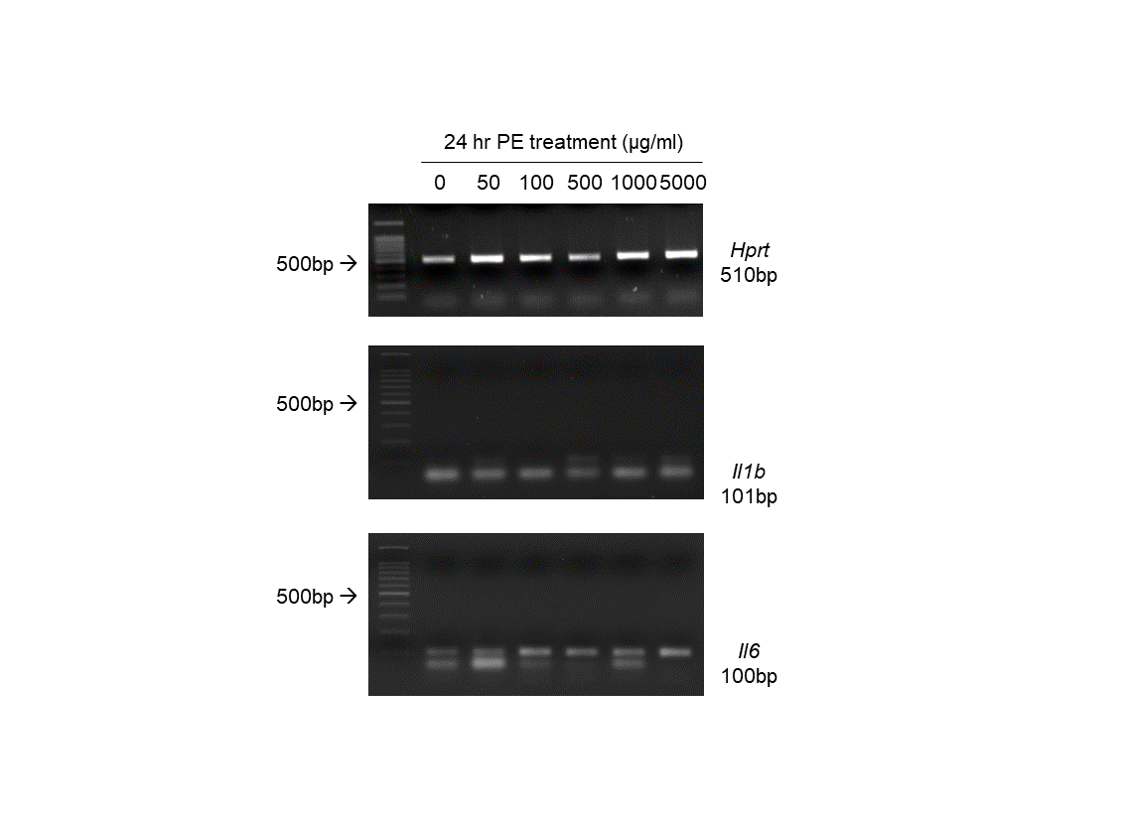

Supplement: S3 Fig — (TIF) [file pone.0301618.s003.tif]

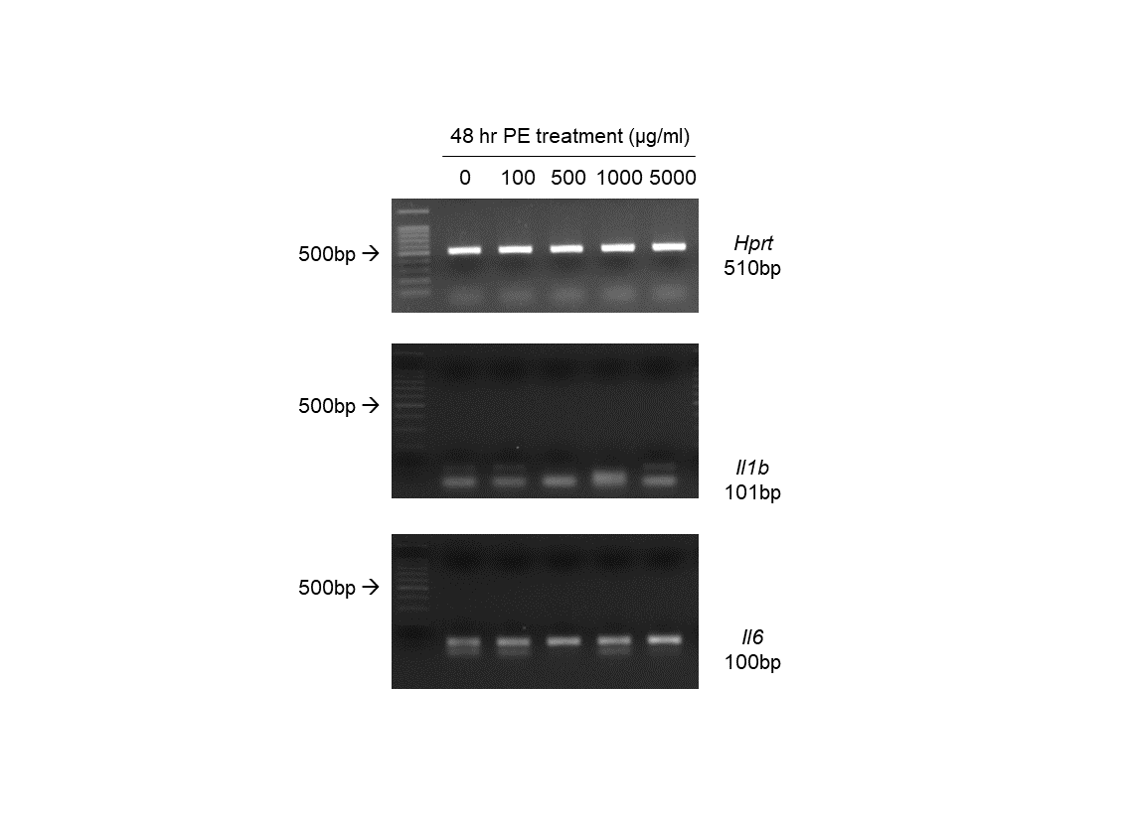

Supplement: S4 Fig — (TIF) [file pone.0301618.s004.tif]

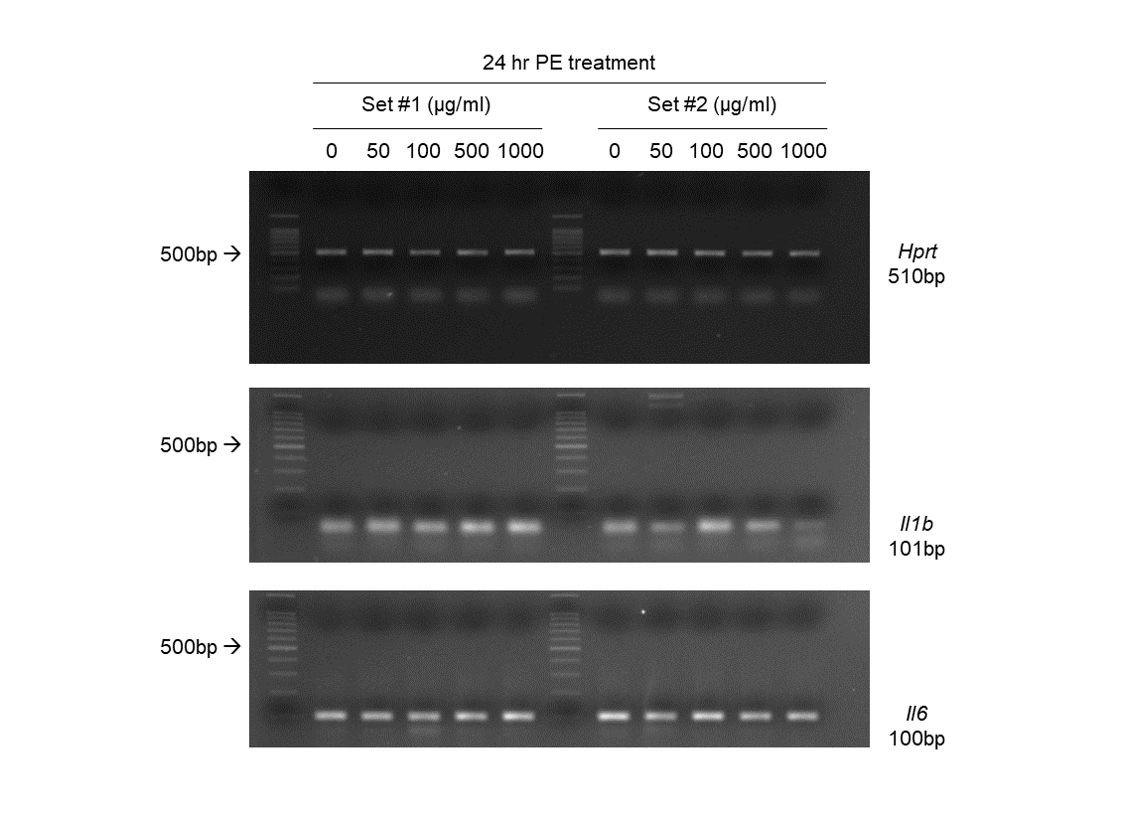

Supplement: S5 Fig — (TIF) [file pone.0301618.s005.tif]

S2 Fig

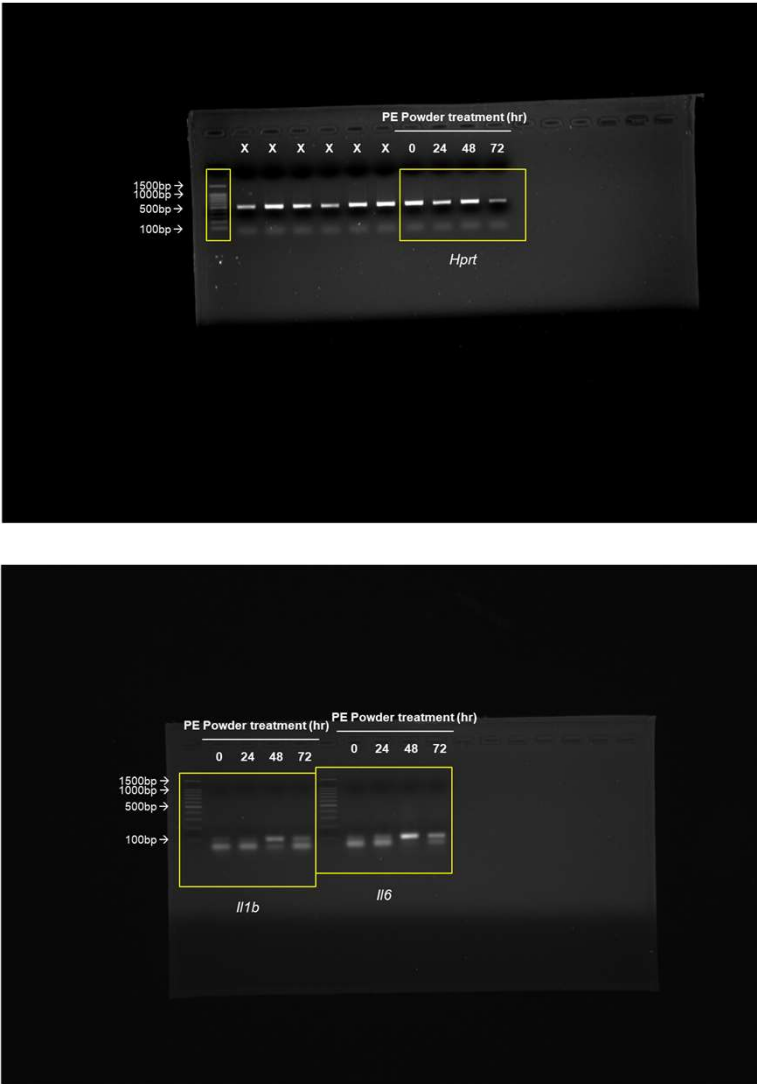

S3 Fig

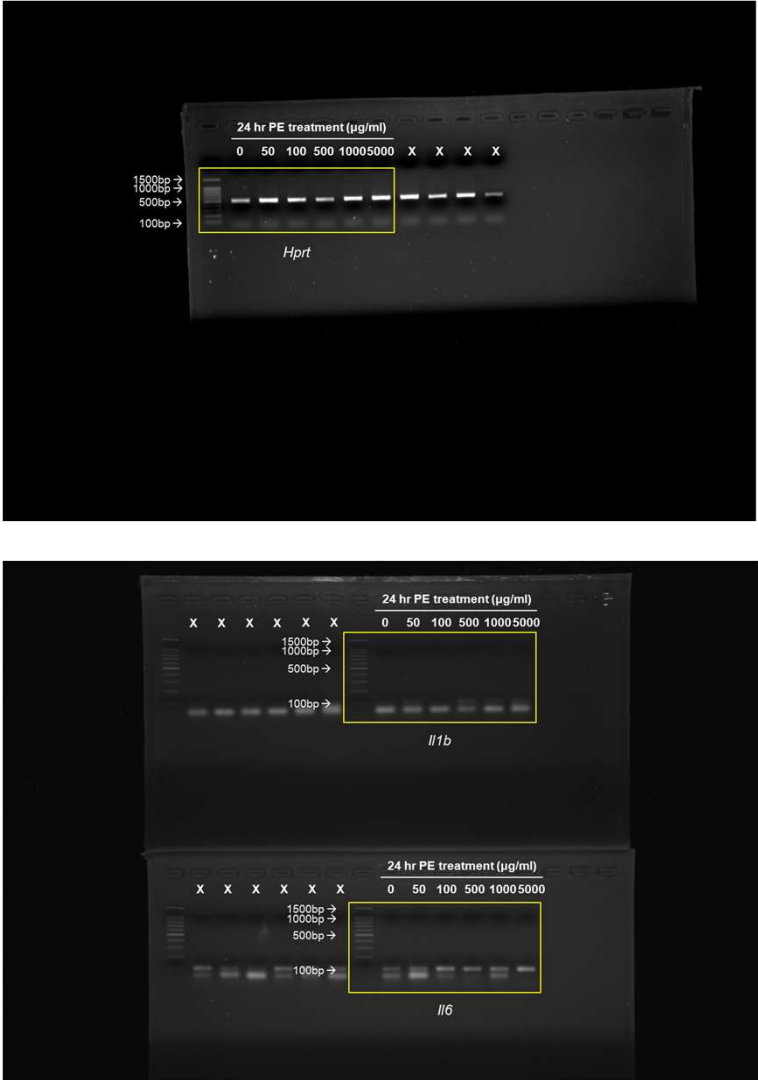

S4 Fig

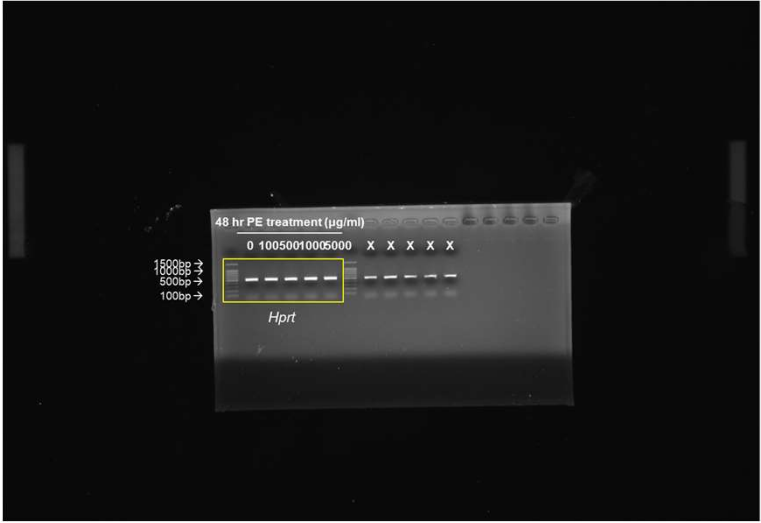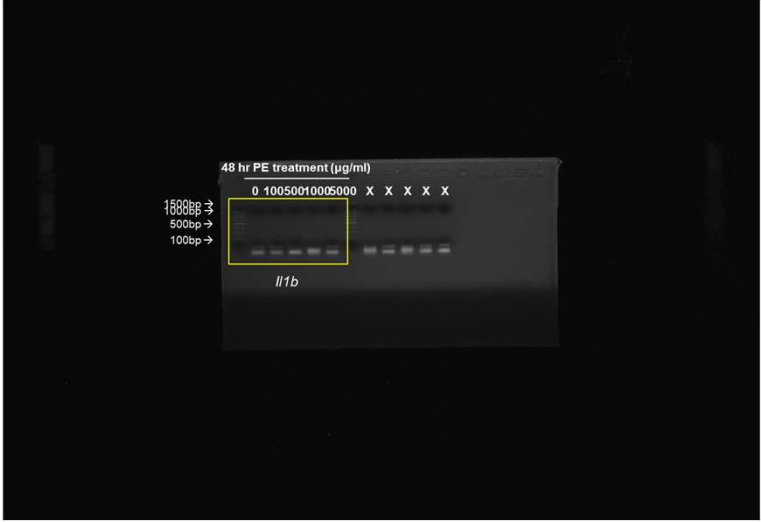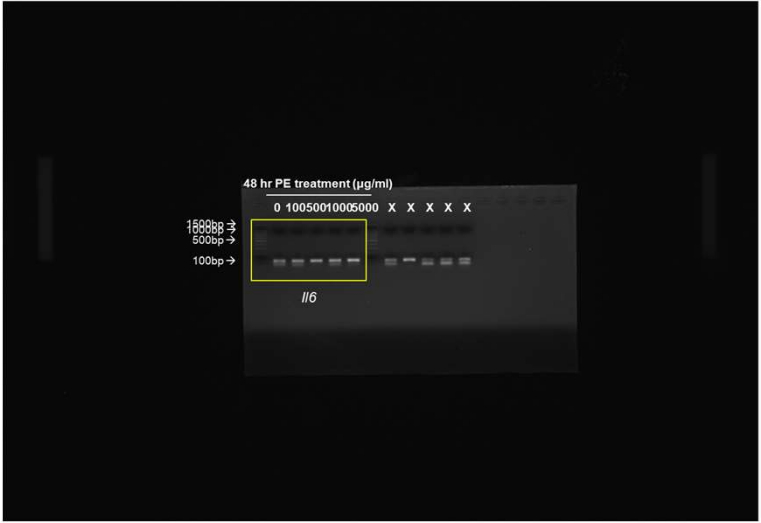

S5 Fig

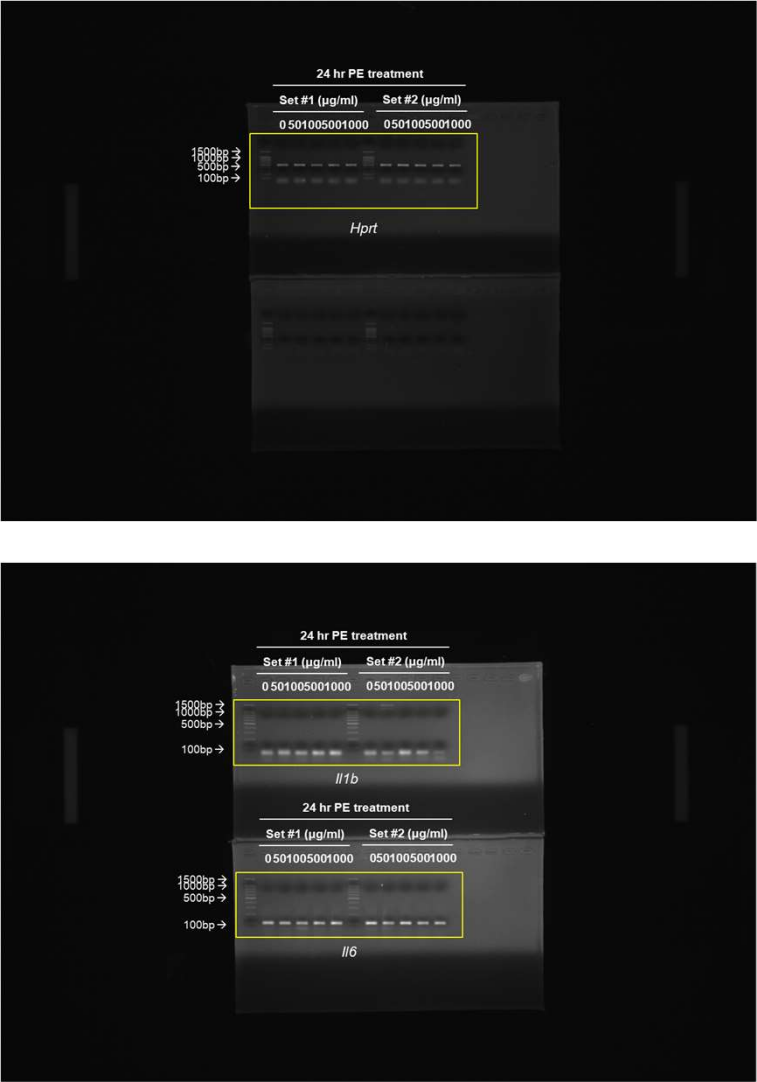

Supplement: S1 Raw images — (PDF) [file pone.0301618.s007.pdf]
