# Supplementary material for: Mitigating polyethylene-mediated periprosthetic tissue inflammation through MEDSAH-grafting
Source: PLoS One. 2024 Jun 6;19(6):e0301618. doi: 10.1371/journal.pone.0301618 (PMC11156361; doi:10.1371/journal.pone.0301618)
Supplement: S1 File — (DOCX) [file pone.0301618.s008.docx]

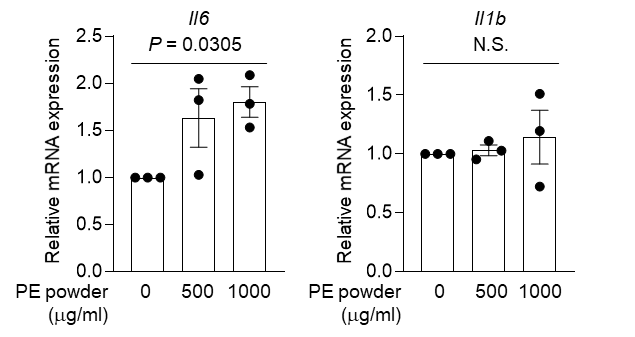


Figure 1. The pro-inflammatory effect of PE powder on J774A.1 macrophages. Relative mRNA expression of inflammatory factors by PE powder doses in J774A.1 cells. Data indicates means ± SEM, one-way ANOVA test.


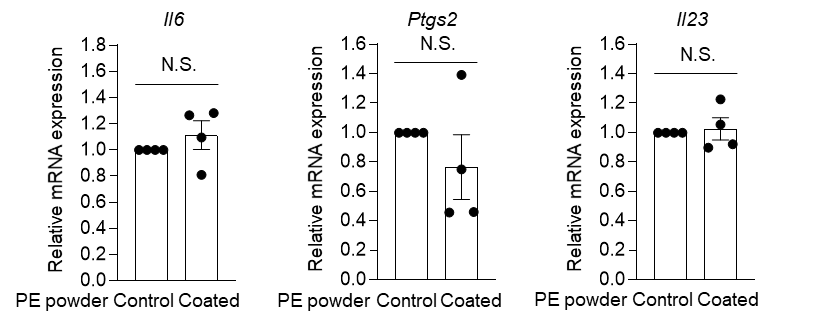


Figure 2. The effect of MEDSAH grafting on PE particles tested in J774A.1 macrophages. Relative mRNA expression of *Il6* and *Ptgs2* between ungrafted PE powder and MEDSAH-coated PE powder (500 μg/ml) in J774.A1 macrophage-like cells. Data indicates means ± SEM, two-tailed Student’s t test.


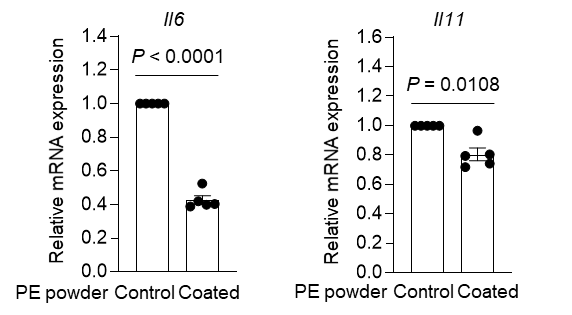


Figure 3. The effect of MEDSAH grafting on IL-6 family cytokine expressions in the primary culture of osteoblasts. Relative mRNA expression of IL-6 family cytokines between ungrafted PE powder and MEDSAH-coated PE powder (500 μg/ml) in primary cultured mouse osteoblasts. Data indicates means ± SEM, two-tailed Student’s t test.


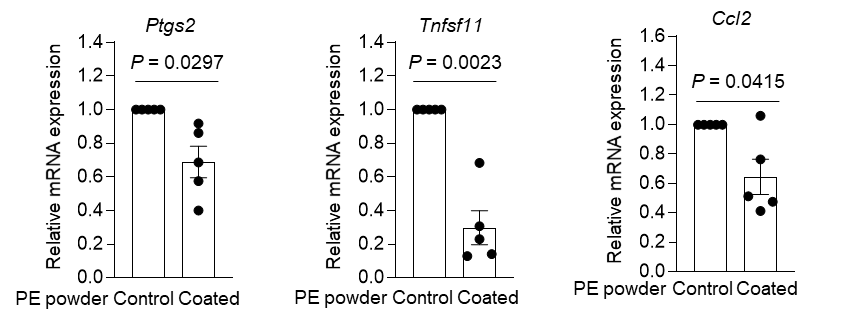


Figure 4. The effect of MEDSAH grafting on other inflammatory factor expressions in the primary culture of osteoblasts. Relative mRNA expression of other osteoblast inflammatory factors between ungrafted PE powder and MEDSAH-coated PE powder in primary cultured mouse osteoblasts. Data indicates means ± SEM, two-tailed Student’s t test.


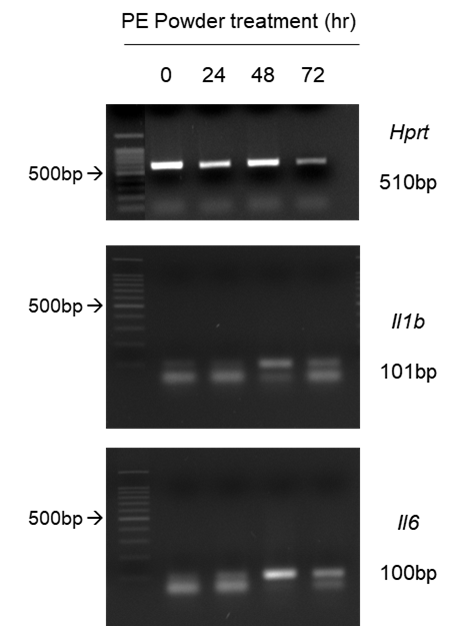


Figure S1. The induction of inflammatory factors in response to the 100 μg/ml ungrafted PE powders at varying time points in J774A.1 cells.


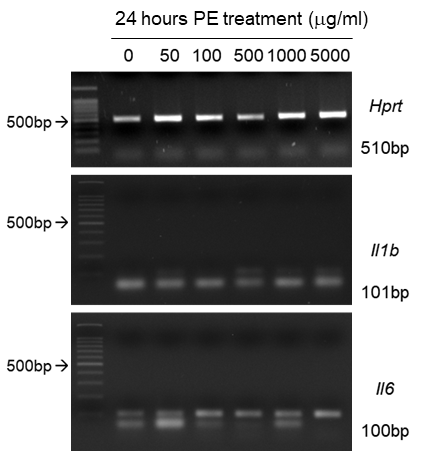


Figure S2. The induction of inflammatory factors in response to varying doses of ungrafted PE powder after 24 hr in J774A.1 cells.


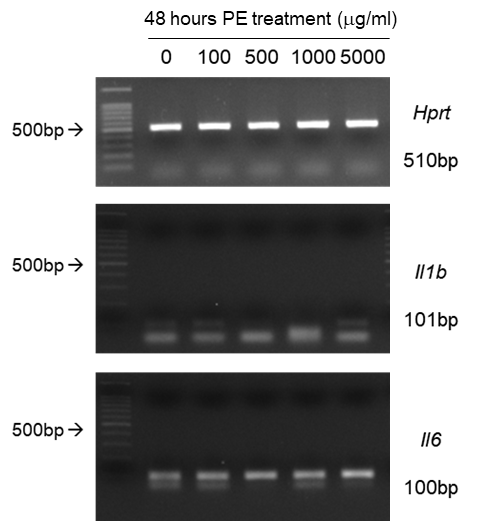


Figure S3. The induction of inflammatory factors in response to varying doses of ungrafted PE powder after 48 hr in J774A.1 cells.


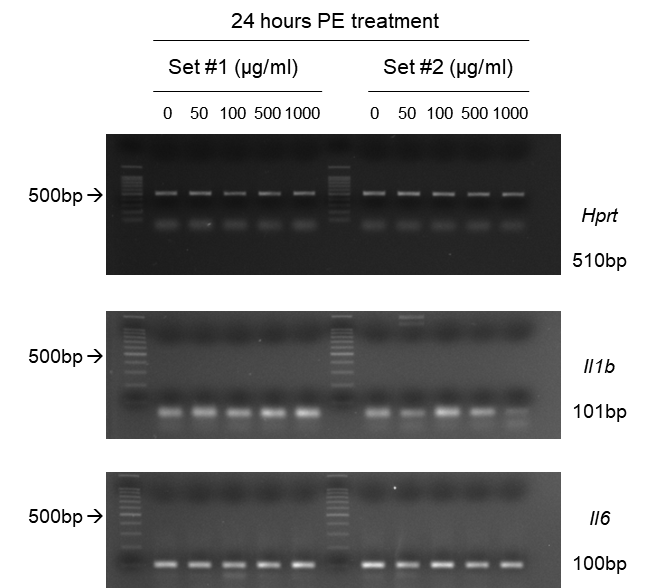


Figure S4. The induction of inflammatory factors in response to the PE powder doses for 24 hr in mouse osteoblasts.


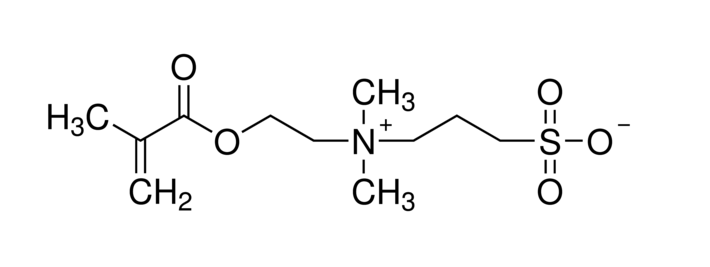


Figure S5. Structural formula of the MEDSAH (2-(Methacryloyloxy)ethyl] dimethyl-(3-sulfopropyl) ammonium hydroxide).
