# Supplementary material for: Mitigating polyethylene-mediated periprosthetic tissue inflammation through MEDSAH-grafting
Source: PLoS One. 2024 Jun 6;19(6):e0301618. doi: 10.1371/journal.pone.0301618 (PMC11156361; doi:10.1371/journal.pone.0301618)
Supplement: Attachment — Submitted filename: Response to Reviewers.docx [file pone.0301618.s009.docx]

**Point-by-point response to the reviewers’ comments**

**Review Comments to the Author**

**Reviewer #1**

**General comment**. The Authors aimed to examine the biological impacts of grafting [2-(methacryloyloxy)ethyl]dimethyl-(3-sulfopropyl)ammonium hydroxide (MEDSAH) to attenuate the inflammatory response induced by polyethylene wear particles.

The topic is interesting and the study is well designed.

Thew paper is well prepared. I have a few minor concerns on its regards.

**[Response]**

We appreciate the reviewer’s careful review of our manuscript. We found all comments to be constructive and helpful, and we have attempted to address your concerns during the revision. We have prepared our detailed point-by-point responses as follows.

**Comment 1.** Introduction should be limited to only relevant information. Also, I would detail further on the possible clinical effects of polyethylene wears in the clinical setting.

**[Response]**

Thank you for your valuable suggestions. To enhance the Introduction’s relevance to our study, we have revised this section by removing two paragraphs. Our research focuses on the biocompatibility benefits of MEDSAH-coating on PE surfaces. Consequently, one paragraph (originally lines 68 to 75) discussing alternative bearing surfaces such as ceramic or metal, and another paragraph (previously lines 85 to 89) detailing different applications of MEDSAH materials, were deemed not directly pertinent and have been removed.

Meanwhile, we have expanded our description on the clinical implications of PE wear in the Introduction. The added text, now in lines 67 to 76, reads: “*In clinical settings, patients undergoing joint replacement surgery are at risk of revision surgery mainly due to loosening and wear [11]. Conventional ultra-high molecular weight polyethylene (UHMWPE) remains one of the most commonly utilized materials for liners or inserts in contemporary total hip and knee arthroplasties [12]. However, long-term observations of conventional PE liners in total hip arthroplasty [13-17] and total knee arthroplasty [18-20] indicated high rates of PE wear and wear-related revisions. Parilla et al. [17] recently identified a marked increase in wear-related revisions, manifested as severe liner wear, osteolysis, implant loosening, and secondary instability, along with a decline in survivorship between the 15-year (87.9%) and 25-year (61.1%) post-operative periods. These findings underscore the clinical significance of PE wear for both the short-term and long-term survivorship of implants.*”

**Comment 2.** Has MEDSAH grafting on PE been studies on a biomechanics point of view? Does this modify the mechanical properties of polyethylene?

**[Response]**

We are grateful for your insightful suggestions. To assess the impact of MEDSAH grafting on the mechanical properties of PE, we constructed the tensile test specimens following the ASTM D638 Type IV geometry and conducted tensile strength test by using the Mechanical Testing device (ITEL-UTM101; SALT Co., Ltd.). MEDSAH-grafted PE displayed a tensile strength of 40.50 ± 1.17 MPa, which did not differ significantly from that of the ungrafted PE of 42.89 ± 1.76 MPa (Fig 2). Consequently, we concluded that the grafting of MEDSAH onto PE does not alter its tensile strength, thus preserving its mechanical properties.

The new data from tensile strength test experiments are presented in Fig. 2 and are described in the Results section under the subheading, ‘*Effect of MEDSAH grafting on PE*’ in lines 176 to 181.

**Fig 2. Tensile strength of ungrafted and MEDSAH-grafted PE.** Tensile strength at break between the tensile test specimens composed of ungrafted PE and MEDSAH-coated PE (left). Tensile strength specimens after the operations on the mechanical testing machine (right). Data indicate means ± SEM; data were compared with two-tailed Student’s t test.

We have added the procedures for this mechanical testing in the Materials and methods section under the subheading of ‘*Tensile test*’, in lines 101 to 107 of the revised manuscript.

**Comment 3.** I would mitigate conclusions as these are not completely supported by results.

**[Response]**

As per your suggestion, we have revised the Discussion section to more closely align with our experimental results. First, we made it explicit that our study was conducted at the *in vitro* level and accordingly moderated our discussion, focusing on the potential clinical advantages associated with the reduced inflammatory response of MEDSAH-grafted PE materials (mainly the 3^rd^ paragraph of the Discussion section in the revised manuscript). Moreover, we entirely removed our speculation on various potential medical device applications utilizing MEDSAH coating, given the current absence of *in vivo* or clinical data (lines 190 to 195 and 211 to 218 in the original manuscript). Through these changes, we aimed to limit our conclusions to those directly supported by our results.

**Comment 4.** Many grammar and syntax errors. Please have the paper checked by a native speaker.

**[Response]**

Thank you for the suggestion. During the revision process, we utilized the professional English editing service, Editage (CACTUS Communications) and had the entire manuscript thoroughly proofread by native English editors. Attached below is a certificate of editing, issued by Editage.

Certificate of editing issued by Editage

**Reviewer #2**

**Comment 1.** 98 … for 30 second —> for 30 seconds

**[Response]**

Thank you for the correction. In accordance with PLOS ONE’s guidelines for the use of time units, we have made the appropriate adjustments. The edited sentence in lines 93 to 95 now reads: “*The raw UHMWPE particles were immersed in an acetone solution containing 10 mg/ml benzophenone for 30 s and then dried to remove the acetone at room temperature.*”

**Comment 2.** 110 … as previously described —> as described above

**[Response]**

Thank you for pointing it out. We acknowledge that our initial description could have been clearer, and have thus revised the text for improved clarity. The modified sentence in lines 115 to 116 now reads: “*Mouse osteoblast isolation and primary culture were performed by using the method previously described by Suh et al. [22].*”

**Comment 3.** 117 … primary cultured mouse osteoblasts passaged three to five times where used for experiments —> Experiments were conducted using primary cultured mouse osteoblasts that had been passed through three to five times.

**[Response]**

We appreciate your correction. We have edited the text in lines 123 to 124 accordingly, which now reads: “*Experiments were conducted using primary cultured mouse osteoblasts that had been passed three to five times.*”

**Journal requirements**

**When submitting your revision, we need you to address these additional requirements.**

**1.** Please ensure that your manuscript meets PLOS ONE's style requirements, including those for file naming. The PLOS ONE style templates can be found at

https://journals.plos.org/plosone/s/file?id=wjVg/PLOSOne_formatting_sample_main_body.pdf and

https://journals.plos.org/plosone/s/file?id=ba62/PLOSOne_formatting_sample_title_authors_affiliations.pdf

**[Response]**

We now have adhered to PLOS ONE’s style requirements and formatting guidelines, making edits including file naming, font size, figure legend disposition, and author affiliations. We utilized the PACE tool to review all figure files, ensuring they comply with PLOS's standards for figures as per the resubmission guidelines.

**2.** We noticed you have some minor occurrence of overlapping text with the following previous publication(s), which needs to be addressed:

Surface grafting of artificial joints with a biocompatible polymer for preventing periprosthetic osteolysis - https://doi.org/10.1038/nmat1233

In your revision ensure you cite all your sources (including your own works), and quote or rephrase any duplicated text outside the methods section. Further consideration is dependent on these concerns being addressed.

**[Response]**

We conducted an ‘Originality Check’ on our manuscript against the specified paper (https://doi.org/10.1038/nmat1233) using *Turnitin* and *Copykiller*. We did identify some textual overlap in the introductory section, where it discussed ‘the current challenges associated with total joint replacement procedures’. We have thoroughly restructured and edited the relevant texts. The modified texts showed no similarities when checked with both software programs. The modified sections are marked in blue in the revised manuscript.

**3.** To comply with PLOS ONE submissions requirements, in your Methods section, please provide additional information regarding the experiments involving animals and ensure you have included details on (1) methods of sacrifice, (2) methods of anesthesia and/or analgesia, and (3) efforts to alleviate suffering.

**[Response]**

We have included additional details in the ‘Cell culture and PE powder treatments’ section of the Materials and methods, between lines 116 and 119. The revised text reads: “*Briefly, mice were anesthetized by isoflurane and decapitated. Calvarial bone tissues were isolated from newborn ICR mice (day 0 to 3) and sequentially digested in 0.25% trypsin-EDTA for 20 min, followed by a solution containing 0.2% type II collagenase (Sigma Aldrich) for 30 min.*”

**4.** Thank you for stating the following in the Acknowledgments Section of your manuscript:

“This work was supported by the Korea Medical Device Development Fund grant funded by Korea government (the Ministry of Science and ICT, the Ministry of Trade, Industry and Energy, the Ministry of Health and Welfare, and the Ministry of Food and Drug Safety; Project Number RS-2020-KD000038), grants from the National Research Foundation of Korea (NRF-2023R1A2C3003864, NRF-2016R1A5A1010764, NRF-2017M3A9D8064193, and NRF-2021R1I1A1A01055626), the Institute for Basic Science from the Ministry of Science, ICT and Future Planning of Korea (IBS-R008-D1), and Suh Kyungbae foundation (SUHF-18010068).”

We note that you have provided funding information that is currently declared in your Funding Statement. However, funding information should not appear in the Acknowledgments section or other areas of your manuscript. We will only publish funding information present in the Funding Statement section of the online submission form.

Please remove any funding-related text from the manuscript and let us know how you would like to update your Funding Statement. Currently, your Funding Statement reads as follows:

“This work was supported by the Korea Medical Device Development Fund grant funded by Korea government (the Ministry of Science and ICT, the Ministry of Trade, Industry and Energy, the Ministry of Health and Welfare, and the Ministry of Food and Drug Safety; Project Number RS-2020-KD000038), grants from the National Research Foundation of Korea (NRF-2023R1A2C3003864, NRF-2016R1A5A1010764, NRF-2017M3A9D8064193, and NRF-2021R1I1A1A01055626), the Institute for Basic Science from the Ministry of Science, ICT and Future Planning of Korea (IBS-R008-D1), and Suh Kyungbae foundation (SUHF-18010068). The funders had no role in study design, data collection and analysis, decision to publish, or preparation of the manuscript.”

Please include your amended statements within your cover letter; we will change the online submission form on your behalf.

**[Response]**

We deleted the informations related to the funding statement from the Acknowledgments section. The revised Acknowledgments section reads: “*All individuals who contributed significantly to the work have been named as authors.*”

Our updated Funding Statement now reads: “*This work was supported by the Korea Medical Device Development Fund grant funded by Korea government (the Ministry of Science and ICT, the Ministry of Trade, Industry and Energy, the Ministry of Health and Welfare, and the Ministry of Food and Drug Safety; Project Number RS-2020-KD000038), grants from the National Research Foundation of Korea (NRF-2023R1A2C3003864 and NRF-2021R1I1A1A01055626), and the Korea Drug Development Fund funded by the Ministry of Science and ICT, the Ministry of Trade, Industry and Energy, and the Ministry of Health and Welfare (RS-2023-00217266). The funders had no role in study design, data collection and analysis, decision to publish, or preparation of the manuscript.*”

The amended statement was also included in our cover letter.

**5.** Please include captions for your Supporting Information files at the end of your manuscript, and update any in-text citations to match accordingly. Please see our Supporting Information guidelines for more information: http://journals.plos.org/plosone/s/supporting-information.

**[Response]**

We have appended captions for the Supporting information files at the end of our manuscript. We updated in-text citations, complying journal’s style (e.g. S1 Fig). Our manuscript contains five supporting figures (S1 Fig to S5 Fig).

**6.** PLOS ONE now requires that authors provide the original uncropped and unadjusted images underlying all blot or gel results reported in a submission’s figures or Supporting Information files. This policy and the journal’s other requirements for blot/gel reporting and figure preparation are described in detail at https://journals.plos.org/plosone/s/figures#loc-blot-and-gel-reporting-requirements and https://journals.plos.org/plosone/s/figures#loc-preparing-figures-from-image-files. When you submit your revised manuscript, please ensure that your figures adhere fully to these guidelines and provide the original underlying images for all blot or gel data reported in your submission. See the following link for instructions on providing the original image data: https://journals.plos.org/plosone/s/figures#loc-original-images-for-blots-and-gels.

In your cover letter, please note whether your blot/gel image data are in Supporting Information or posted at a public data repository, provide the repository URL if relevant, and provide specific details as to which raw blot/gel images, if any, are not available. Email us at plosone@plos.org if you have any questions.

**[Response]**

We have presented uncropped and unadjusted images in S2 to S5 Fig, which display the molecular weight markers ranging from 100 to 10,000 nucleotide in length.

**7.** Please review your reference list to ensure that it is complete and correct. If you have cited papers that have been retracted, please include the rationale for doing so in the manuscript text, or remove these references and replace them with relevant current references. Any changes to the reference list should be mentioned in the rebuttal letter that accompanies your revised manuscript. If you need to cite a retracted article, indicate the article’s retracted status in the References list and also include a citation and full reference for the retraction notice.

**[Response]**

We have ensured that our manuscript does not include any citations of retracted articles. This includes all 32 references from our original submission and the eight references we incorporated during the revision process.

The modifications made to the reference list are outlined below:

Deleted References: Refs. 11-16, 18-20, 31-35 in the original manuscript. These deletions were made in accordance with Reviewer #1’s comments 1 and 3, which suggested shortening the text by removing less relevant citations from the Introduction and Discussion sections.

Added References: In response to the request by Reviewer #1 (comment 1), we added references 11-15, 17, 19, and 20 to the revised manuscript to enrich the discussion on the potential clinical impact of PE wear in a clinical setting.
